# Chlorido(pyridine-κ*N*)(5,10,15,20-tetra­phenyl­porphyrinato-κ^4^ *N*)cobalt(III) chloro­form hemisolvate

**DOI:** 10.1107/S1600536812032564

**Published:** 2012-07-25

**Authors:** Yassin Belghith, Jean-Claude Daran, Habib Nasri

**Affiliations:** a Département de Chimie, Faculté des Sciences de Monastir, Université de Monastir, Avenue de l’environnement, 5019 Monastir, Tunisia; b Laboratoire de Chimie de Coordination, CNRS UPR 8241, 205 route de Norbonne, 31077 Toulouse, Cedex 04, France

## Abstract

In the title complex, [CoCl(C_44_H_28_N_4_)(C_5_H_5_N)]·0.5CHCl_3_ or [Co^III^(TPP)Cl(py)]·0.5CHCl_3_ (where TPP is the dianion of tetra­phenyl­porphyrin and py is pyridine), the average equatorial cobalt–pyrrole N atom bond length (Co—N_p_) is 1.958 (7) Å and the axial Co—Cl and Co—N_py_ distances are 2.2339 (6) and 1.9898 (17) Å, respectively. The tetra­phenyl­porphyrinate dianion exhibits an important nonplanar conformation with major ruffling and saddling distortions. In the crystal, mol­ecules are linked *via* weak C—H⋯π inter­actions. In the difference Fourier map, a region of highly disordered electron density was estimated using the SQUEEZE routine [*PLATON*; Spek (2009 ▶), *Acta Cryst.* D**65**, 148–155] to be equivalent to one half-mol­ecule of CHCl_3_ per mol­ecule of the complex.

## Related literature

For general background on cobalt porphyrin species and their applications, see: Sanders *et al.* (2000 ▶). For the synthesis of Co(II) tetra­phenyl­porphyrin, see: Madure & Scheidt (1976 ▶). For metalloporphyrins used as biomimetic models for haemoproteines, see: Dhifet *et al.* (2010 ▶); Mansour *et al.* (2010 ▶). For the structures of related compounds, see: Ali *et al.* (2011 ▶); Goodwin *et al.* (2001 ▶); Hodgson *et al.* (2002 ▶); Iimuna *et al.* (1988 ▶); Jentzen *et al.* (1997 ▶); Konarev *et al.* (2003 ▶); Mikolaiski *et al.* (1989 ▶); Sakurai *et al.* (1976 ▶); Shirazi & Goff (1982 ▶); Toronto *et al.* (1998 ▶). For the Cambridge Structural Database, see: Allen (2002 ▶). For details of the SQUEEZE routine in *PLATON*, see: Spek (2009 ▶).
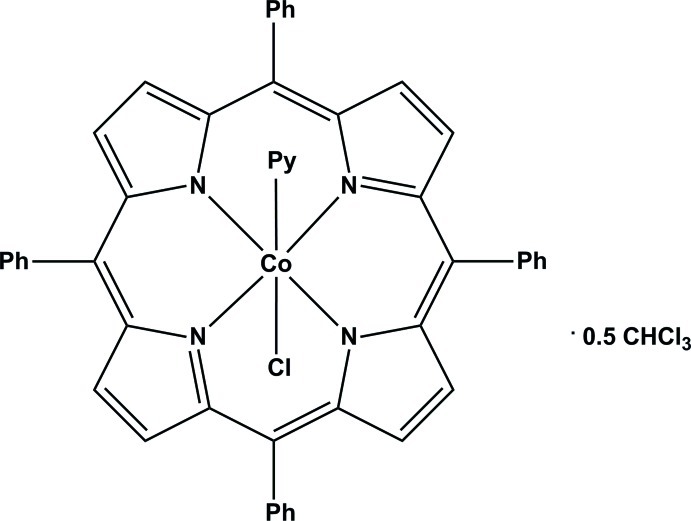


## Experimental

### 

#### Crystal data

[CoCl(C_44_H_28_N_4_)(C_5_H_5_N)]·0.5CHCl_3_
*M* *_r_* = 845.90
Monoclinic, 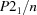

*a* = 13.0467 (3) Å
*b* = 23.4240 (7) Å
*c* = 14.3264 (5) Å
β = 103.541 (3)°
*V* = 4256.5 (2) Å^3^
*Z* = 4
Mo *K*α radiation
μ = 0.60 mm^−1^
*T* = 180 K
0.45 × 0.37 × 0.36 mm

#### Data collection

Oxford Xcalibur Sapphire2 diffractometer with a large Be window
Absorption correction: multi-scan (*CrysAlis PRO*; Agilent, 2010 ▶) *T*_min_ = 0.802, *T*_max_ = 0.804
43618 measured reflections
8690 independent reflections
7213 reflections with *I* > 2σ(*I*)
*R*_int_ = 0.035

#### Refinement

*R*[*F* ^2^ > 2σ(*F* ^2^)] = 0.039
*wR*(*F* ^2^) = 0.108
*S* = 1.07
8690 reflections
505 parameters
H-atom parameters constrained
Δρ_max_ = 0.30 e Å^−3^
Δρ_min_ = −0.37 e Å^−3^

### 

Data collection: *CrysAlis PRO* (Agilent, 2010 ▶); cell refinement: *CrysAlis PRO*; data reduction: *CrysAlis PRO*; program(s) used to solve structure: *SIR2004* (Burla *et al.*, 2005 ▶; program(s) used to refine structure: *SHELXL97* (Sheldrick, 2008 ▶); molecular graphics: *ORTEPIII* (Burnett & Johnson, 1996 ▶) and *ORTEP-3* (Farrugia, 1997 ▶); software used to prepare material for publication: *SHELXL97*.

## Supplementary Material

Crystal structure: contains datablock(s) I, global. DOI: 10.1107/S1600536812032564/su2453sup1.cif

Structure factors: contains datablock(s) I. DOI: 10.1107/S1600536812032564/su2453Isup2.hkl

Additional supplementary materials:  crystallographic information; 3D view; checkCIF report

## Figures and Tables

**Table 1:** Hydrogen-bond geometry (Å, °) *Cg*2, *Cg*3, *Cg*6, *Cg*9, *Cg*11 and *Cg*12 are the centroids of the N2/C6–C9, N3/C11–C14, Co/N1/C4–C6/N2, N5/C45–C49, C27–C32 and C33–C38 rings, respectively.

*D*—H⋯*A*	*D*—H	H⋯*A*	*D*⋯*A*	*D*—H⋯*A*
C24—H24⋯*Cg*3^i^	0.95	2.79	3.543 (3)	137
C28—H28⋯*Cg*9^ii^	0.95	2.79	3.735 (3)	172
C35—H35⋯*Cg*2^iii^	0.95	2.87	3.736 (2)	152
C38—H38⋯*Cg*11^iv^	0.95	2.98	3.861 (3)	156
C42—H42⋯*Cg*12^v^	0.95	2.75	3.574 (3)	146
C49—H49⋯*Cg*6	0.95	2.35	2.931 (3)	119

Symmetry codes: (i) 
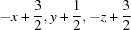
; (ii) 
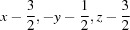
; (iii) 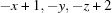; (iv) 
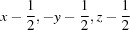
; (v) 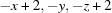.

**Fig. 1.**
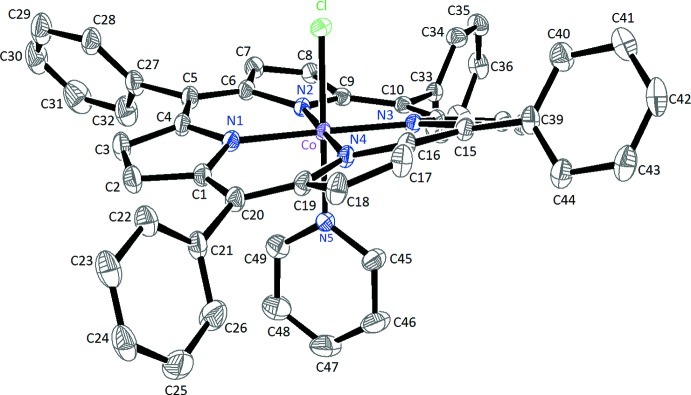
A view of the molecular structure of the title molecule with the atom-numbering. Displacement ellipsoids are drawn at 50% and H atoms have been omitted for clarity.

**Fig. 2.**
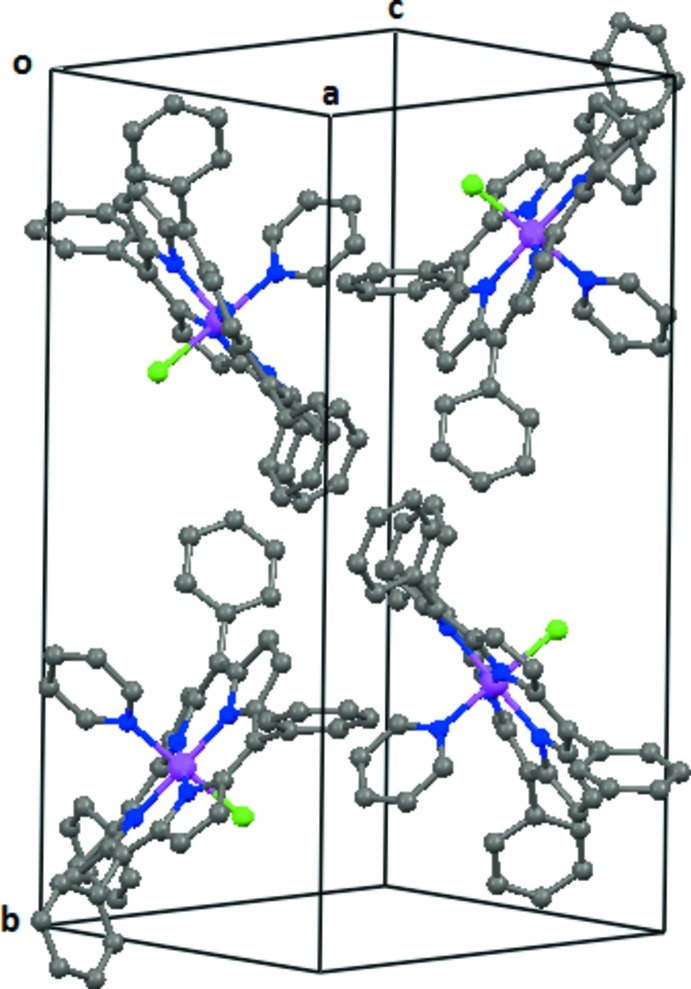
A view perpendicular to (101) of the crystal packing of the title compound. The H atoms have been omitted for clarity.

## supplementary crystallographic information

### Comment

As part of a systematic investigation of metalloporphyrins used as biomimetic models for hemoproteines, several iron and cobalt porphyrin complexes has been characterized by our group (Dhifet *et al.*, 2010; Mansour *et al.*, 2010). Cobalt porphyrin species and their applications have been discussed by (Sanders *et al.*, 2000). The chlorido cobalt(III) porphyrin derivative [Co^III^(TPP)Cl] (TPP is the dianion of the tetraphenylporphyrin) has been known for many decades (Sakurai *et al.*, 1976) but to the best of our knowledge no crystal structure of a mono or dichlorido Co(III) metalloporphyrin is reported to date. This is also the case for mono-pyridine or bis-pyridine Co(III) porphyrins. A search of Cambridge Structural Database (CSD, version 5.31; Allen, 2002) reveals: (i) only two mixed-axial ligands Cl–Co(III)–*X* (*X* = H_2_O or EtOH) porphyrin structures; (CSD refcodes GAMTAP; Iimuna *et al.*, 1988 and PUCNUW; Toronto *et al.*, 1998, respectively), (ii) three mixed-axial ligands py–Co(III)–*L* (*L* is a monodentate ligand) porphyrin derivatives: the [Co^III^(TpivPP)Br(py)] (refcode LANTIE; Hodgson *et al.*, 2002), the [Co^III^(TPP)(NO_2_)(py)] (refcode YEQMIQ; Goodwin *et al.*, 2001) and the [Co^III^(TPP)(py)(C_3_H_5_O_2_)] (refcode KEBMIN; Mikolaiski *et al.*, 1989).

Concerning the ^1^H NMR of cobalt metalloporphyrins, it has been noticed that the paramagnetic starting material [Co^II^(TPP)] species (with the ground state configuration 3d^7^) presents down-field chemical shifts of the H_β_-pyrrole protons [H_β__-_pyrr = 15.75 p.p.m.]. For the diamagnetic cobalt(III) porphyrin derivatives (with the ground state configuration 3d^6^), the β-pyrrole protons resonate in the normal regions of the free base TPP porphyrin [8.1 p.p.m. < δ(H_β__-_pyrr) < 9.4 p.p.m.] (Shirazi Goff, 1982). Complex (I) presents a peak at 9.13 p.p.m. attributed to the β-pyrr protons, which is an indication that our derivative is a diamagnetic cobalt(III) *meso*-porphyrin species.

We report herein on the molecular structure of the title compound, a mixed-ligand py–Co(III)–Cl tetraphenylporphyrin species [Co^III^(TPP)Cl(py)]. In this complex, the cobalt is coordinated to the four N atoms of the porphyrin ring, the chloride ion and the nitrogen atom of the pyridine axial ligand (Fig. 1). The axial Co—Cl bond length [2.2339 (6) Å] is similar to those in the two related species: [Co^III^(TPP)Cl(H_2_O)] [Co—Cl = 2.216 (1) Å] and [Co^III^(TMCP)Cl(OHCH_2_CH_3_)] (TMCP is the αβαβ-tetra-methylchiroporphyrin) [Co—Cl = 2.211 (2) Å] complexes. The Co—N_p_ distance [1.989 (2) Å] is slightly shorter than those of the three related derivatives [Co^III^(Porph)(*L*)(py)] where *L* = Br, C_3_H_5_O_2_ and NO_2_ [2.00 (2)–2.043 (7) Å]. It has been noticed that there is a relationship between the ruffling of the porphyrinato core and the mean equatorial Co—N_p_ distance; the porphyrinato core is ruffled as the Co—N_p_ distance decreases (Iimuna *et al.*, 1988). Thus, for the very ruffled structure [Co^II^(TPP)] (Konarev *et al.*, 2003) the Co—N_p_ bond length value is 1.923 (4) Å while the practically planar porphyrin core of the ion complex [Co^III^(OEP)(NO_2_)_2_]^-^ (OEP is the octaethylporphyrin; Ali *et al.*, 2011) presents a Co—N_p_ distance of 1.988 (2) Å. Therefore, the Co—N_p_ distance in the title complex [1.958 (2) Å] is normal for a cobalt ruffled TPP species. On the other hand Normal Structural Decomposition (NSD) calculations (Jentzen *et al.*, 1997) confirm the unusually important deformation of the porphyrin core with a major ruffling and saddling distortions of 52% and 39%, respectively.

The crystal packing is stabilized by weak C—H······π interactions (Table 1 and Fig. 2).

### Experimental

[Co^II^(TPP)] (100 mg, 0.149 mmol) (Madure & Scheidt 1976) and (150 mg, 1.441 mmol) of NaHSO_3_ and (18 ml, 0.223 mmol) of pyridine in 25 ml of chloroform were stirred overnight at room temperature. The color of the solution turns from red–orange to dark–red and the final product is the title complex [Co^III^(TPP)Cl(py)].0.5(CHCl_3_). This means that the NaHSO_3_ reagent did not react with [Co^II^(TPP)] and that the Cl^-^ anion comes from the chlorinated solvent. This is expected given the high affinity of chloride for the cobalt ion. Crystals of the title compound were grown by diffusion of hexanes into a chloroform solution of the title compound.

### Refinement

Hydrogen atoms were placed in calculated positions and refined as riding atoms: C—H = 0.95 Å with *U*_iso_(H) = 1.2 *U*_eq_(C). In a final difference Fourier map highly disordered electron density occupying two cavities of *ca* 389 Å^3^ each was observed. This residual electron density was difficult to model and therefore, the SQUEEZE routine in *PLATON* (Spek, 2009) was used to eliminate this contribution of the electron density in the solvent region from the intensity data. The solvent-free model was employed for the final refinement. It was estimated that each cavity contains 59 electrons which corresponds to a solvent molecule of chloroform as suggested by chemical analysis, or half a molecule of CHCl_3_ per molecule of complex.

### Crystal data

**Table:**

[CoCl(C_44_H_28_N_4_)(C_5_H_5_N)]·0.5CHCl_3_	*F*(000) = 1740
*M**_r_* = 845.90	*D*_x_ = 1.315 Mg m^−^^3^
Monoclinic, *P*2_1_/*n*	Mo *K*α radiation, λ = 0.71073 Å
Hall symbol: -P 2yn	Cell parameters from 25194 reflections
*a* = 13.0467 (3) Å	θ = 3.0–26.4°
*b* = 23.4240 (7) Å	µ = 0.60 mm^−^^1^
*c* = 14.3264 (5) Å	*T* = 180 K
β = 103.541 (3)°	Prism, dark purple
*V* = 4256.5 (2) Å^3^	0.45 × 0.37 × 0.36 mm
*Z* = 4	

### Data collection

**Table:**

Oxford Xcalibur Sapphire2 diffractometer with a large Be window	8690 independent reflections
Radiation source: fine-focus sealed tube	7213 reflections with *I* > 2σ(*I*)
Graphite monochromator	*R*_int_ = 0.035
Detector resolution: 8.2632 pixels mm^-1^	θ_max_ = 26.4°, θ_min_ = 3.0°
ω scans	*h* = −16→16
Absorption correction: multi-scan (*CrysAlis PRO*; Agilent, 2010)	*k* = −29→29
*T*_min_ = 0.802, *T*_max_ = 0.804	*l* = −17→17
43618 measured reflections	

### Refinement

**Table:**

Refinement on *F*^2^	Primary atom site location: structure-invariant direct methods
Least-squares matrix: full	Secondary atom site location: difference Fourier map
*R*[*F*^2^ > 2σ(*F*^2^)] = 0.039	Hydrogen site location: inferred from neighbouring sites
*wR*(*F*^2^) = 0.108	H-atom parameters constrained
*S* = 1.07	*w* = 1/[σ^2^(*F*_o_^2^) + (0.0587*P*)^2^ + 2.0185*P*] where *P* = (*F*_o_^2^ + 2*F*_c_^2^)/3
8690 reflections	(Δ/σ)_max_ = 0.001
505 parameters	Δρ_max_ = 0.30 e Å^−^^3^
0 restraints	Δρ_min_ = −0.37 e Å^−^^3^

### Special details

**Table:**

Experimental. Absorption correction: Empirical absorption correction using spherical harmonics, implemented in SCALE3 ABSPACK scaling algorithm (CrysAlisPro; Agilent, 2010).
Geometry. All s.u.'s (except the s.u. in the dihedral angle between two l.s. planes) are estimated using the full covariance matrix. The cell s.u.'s are taken into account individually in the estimation of s.u.'s in distances, angles and torsion angles; correlations between s.u.'s in cell parameters are only used when they are defined by crystal symmetry. An approximate (isotropic) treatment of cell s.u.'s is used for estimating s.u.'s involving l.s. planes.
Refinement. Refinement of *F*^2^ against ALL reflections. The weighted *R*-factor *wR* and goodness of fit *S* are based on *F*^2^, conventional *R*-factors *R* are based on *F*, with *F* set to zero for negative *F*^2^. The threshold expression of *F*^2^ > σ(*F*^2^) is used only for calculating *R*-factors(gt) *etc*. and is not relevant to the choice of reflections for refinement. *R*-factors based on *F*^2^ are statistically about twice as large as those based on *F*, and *R*-factors based on ALL data will be even larger.

### Fractional atomic coordinates and isotropic or equivalent isotropic displacement parameters (Å^2^)

**Table:**

	*x*	*y*	*z*	*U*_iso_*/*U*_eq_	
Co	0.653649 (18)	0.198422 (10)	0.876228 (18)	0.01771 (8)	
Cl	0.57928 (4)	0.13891 (2)	0.75784 (4)	0.02806 (12)	
N1	0.56392 (12)	0.26163 (7)	0.81419 (12)	0.0223 (3)	
N2	0.54701 (12)	0.17807 (7)	0.94669 (11)	0.0206 (3)	
N3	0.74480 (11)	0.13656 (7)	0.93931 (11)	0.0190 (3)	
N4	0.75900 (12)	0.21811 (7)	0.80496 (12)	0.0210 (3)	
N5	0.72264 (13)	0.25204 (7)	0.97968 (12)	0.0235 (3)	
C1	0.59560 (15)	0.30730 (8)	0.76759 (15)	0.0249 (4)	
C2	0.51268 (16)	0.34851 (9)	0.74310 (17)	0.0335 (5)	
H2	0.5156	0.3841	0.7121	0.040*	
C3	0.42995 (17)	0.32728 (9)	0.77237 (18)	0.0352 (5)	
H3	0.3625	0.3446	0.7640	0.042*	
C4	0.46209 (15)	0.27343 (9)	0.81880 (15)	0.0261 (4)	
C5	0.40233 (15)	0.24155 (9)	0.86779 (16)	0.0272 (4)	
C6	0.44397 (15)	0.19627 (8)	0.92754 (15)	0.0233 (4)	
C7	0.38706 (15)	0.16393 (9)	0.98388 (15)	0.0270 (4)	
H7	0.3140	0.1670	0.9819	0.032*	
C8	0.45596 (15)	0.12847 (9)	1.03985 (15)	0.0252 (4)	
H8	0.4414	0.1028	1.0865	0.030*	
C10	0.64470 (14)	0.10443 (8)	1.05469 (13)	0.0205 (4)	
C11	0.73416 (14)	0.10587 (8)	1.01807 (13)	0.0198 (4)	
C12	0.82315 (15)	0.06912 (8)	1.04972 (14)	0.0228 (4)	
H12	0.8363	0.0451	1.1047	0.027*	
C13	0.88449 (15)	0.07503 (8)	0.98695 (14)	0.0230 (4)	
H13	0.9485	0.0554	0.9884	0.028*	
C14	0.83511 (14)	0.11654 (8)	0.91746 (14)	0.0203 (4)	
C15	0.87563 (14)	0.13505 (8)	0.84102 (14)	0.0229 (4)	
C16	0.83825 (14)	0.18342 (9)	0.78865 (15)	0.0238 (4)	
C17	0.88598 (16)	0.20959 (10)	0.71885 (16)	0.0320 (5)	
H17	0.9389	0.1934	0.6914	0.038*	
C18	0.84160 (16)	0.26142 (10)	0.69963 (16)	0.0320 (5)	
H18	0.8603	0.2897	0.6590	0.038*	
C19	0.76113 (15)	0.26602 (9)	0.75165 (15)	0.0249 (4)	
C20	0.69111 (16)	0.31156 (9)	0.74164 (15)	0.0264 (4)	
C21	0.72025 (15)	0.36591 (9)	0.70021 (17)	0.0297 (5)	
C22	0.67501 (19)	0.38346 (10)	0.60764 (17)	0.0353 (5)	
H22	0.6233	0.3603	0.5672	0.042*	
C23	0.7047 (2)	0.43488 (10)	0.57344 (19)	0.0410 (6)	
H23	0.6728	0.4468	0.5099	0.049*	
C24	0.77946 (19)	0.46849 (10)	0.6304 (2)	0.0428 (6)	
H24	0.7990	0.5038	0.6070	0.051*	
C25	0.8261 (2)	0.45096 (12)	0.7216 (2)	0.0551 (8)	
H25	0.8788	0.4739	0.7613	0.066*	
C26	0.7966 (2)	0.39973 (12)	0.7561 (2)	0.0492 (7)	
H26	0.8298	0.3878	0.8193	0.059*	
C27	0.29171 (16)	0.25999 (9)	0.86365 (18)	0.0333 (5)	
C28	0.21741 (17)	0.26194 (10)	0.7771 (2)	0.0399 (6)	
H28	0.2367	0.2512	0.7196	0.048*	
C29	0.11497 (19)	0.27942 (11)	0.7738 (2)	0.0525 (8)	
H29	0.0647	0.2809	0.7140	0.063*	
C30	0.0864 (2)	0.29440 (12)	0.8556 (3)	0.0603 (9)	
H30	0.0159	0.3059	0.8529	0.072*	
C31	0.1585 (2)	0.29308 (13)	0.9420 (3)	0.0613 (9)	
H31	0.1378	0.3036	0.9989	0.074*	
C32	0.2622 (2)	0.27640 (11)	0.9467 (2)	0.0474 (6)	
H32	0.3125	0.2763	1.0066	0.057*	
C33	0.63738 (14)	0.06207 (8)	1.13095 (14)	0.0221 (4)	
C34	0.60805 (16)	0.00627 (9)	1.10584 (16)	0.0287 (4)	
H34	0.6007	−0.0059	1.0413	0.034*	
C35	0.58935 (17)	−0.03197 (9)	1.17365 (17)	0.0338 (5)	
H35	0.5676	−0.0699	1.1554	0.041*	
C36	0.60237 (18)	−0.01485 (10)	1.26792 (17)	0.0364 (5)	
H36	0.5884	−0.0407	1.3145	0.044*	
C37	0.6355 (2)	0.03952 (11)	1.29402 (17)	0.0397 (6)	
H37	0.6468	0.0509	1.3593	0.048*	
C38	0.65278 (18)	0.07817 (10)	1.22583 (16)	0.0323 (5)	
H38	0.6753	0.1159	1.2446	0.039*	
C39	0.96499 (15)	0.10293 (9)	0.81744 (15)	0.0259 (4)	
C40	0.94875 (18)	0.04766 (10)	0.78297 (17)	0.0348 (5)	
H40	0.8823	0.0298	0.7782	0.042*	
C41	1.0294 (2)	0.01821 (11)	0.75534 (19)	0.0420 (6)	
H41	1.0174	−0.0194	0.7305	0.050*	
C42	1.12631 (19)	0.04332 (11)	0.76376 (18)	0.0404 (6)	
H42	1.1809	0.0233	0.7440	0.048*	
C43	1.14398 (18)	0.09762 (11)	0.80098 (18)	0.0388 (6)	
H43	1.2115	0.1146	0.8086	0.047*	
C44	1.06373 (16)	0.12744 (10)	0.82726 (17)	0.0326 (5)	
H44	1.0763	0.1650	0.8522	0.039*	
C45	0.82727 (17)	0.25121 (10)	1.01486 (17)	0.0335 (5)	
H45	0.8677	0.2231	0.9919	0.040*	
C46	0.8785 (2)	0.28936 (12)	1.08273 (19)	0.0456 (6)	
H46	0.9527	0.2871	1.1069	0.055*	
C47	0.8210 (2)	0.33068 (12)	1.1151 (2)	0.0542 (7)	
H47	0.8548	0.3580	1.1611	0.065*	
C48	0.7130 (2)	0.33191 (12)	1.0799 (2)	0.0540 (7)	
H48	0.6713	0.3599	1.1017	0.065*	
C49	0.6669 (2)	0.29215 (10)	1.01310 (18)	0.0380 (5)	
H49	0.5926	0.2930	0.9894	0.046*	
C9	0.55584 (15)	0.13662 (8)	1.01593 (14)	0.0214 (4)	

### Atomic displacement parameters (Å^2^)

**Table:**

	*U*^11^	*U*^22^	*U*^33^	*U*^12^	*U*^13^	*U*^23^
Co	0.01456 (13)	0.01685 (14)	0.02065 (14)	0.00084 (9)	0.00195 (9)	0.00394 (10)
Cl	0.0259 (2)	0.0279 (3)	0.0271 (3)	−0.00221 (19)	−0.00032 (19)	−0.0017 (2)
N1	0.0166 (7)	0.0216 (8)	0.0276 (9)	0.0002 (6)	0.0028 (6)	0.0048 (7)
N2	0.0174 (7)	0.0200 (8)	0.0235 (8)	0.0014 (6)	0.0031 (6)	0.0014 (7)
N3	0.0159 (7)	0.0187 (8)	0.0217 (8)	0.0000 (6)	0.0027 (6)	0.0034 (6)
N4	0.0169 (7)	0.0215 (8)	0.0236 (8)	0.0023 (6)	0.0026 (6)	0.0066 (7)
N5	0.0226 (8)	0.0202 (8)	0.0260 (9)	−0.0007 (6)	0.0023 (7)	0.0024 (7)
C1	0.0221 (9)	0.0216 (10)	0.0291 (11)	0.0028 (7)	0.0020 (8)	0.0060 (8)
C2	0.0280 (11)	0.0250 (11)	0.0480 (14)	0.0064 (8)	0.0098 (10)	0.0139 (10)
C3	0.0255 (10)	0.0281 (12)	0.0522 (14)	0.0091 (9)	0.0093 (10)	0.0128 (10)
C4	0.0180 (9)	0.0245 (10)	0.0343 (11)	0.0047 (8)	0.0029 (8)	0.0065 (9)
C5	0.0194 (9)	0.0250 (10)	0.0367 (12)	0.0039 (8)	0.0056 (8)	0.0050 (9)
C6	0.0174 (9)	0.0236 (10)	0.0291 (10)	0.0024 (7)	0.0056 (8)	0.0010 (8)
C7	0.0207 (9)	0.0283 (11)	0.0343 (11)	0.0010 (8)	0.0109 (8)	0.0010 (9)
C8	0.0247 (10)	0.0242 (10)	0.0289 (11)	−0.0003 (8)	0.0105 (8)	0.0024 (8)
C10	0.0232 (9)	0.0185 (9)	0.0190 (9)	−0.0016 (7)	0.0032 (7)	0.0005 (7)
C11	0.0195 (9)	0.0168 (9)	0.0215 (9)	−0.0009 (7)	0.0015 (7)	0.0023 (7)
C12	0.0227 (9)	0.0212 (10)	0.0224 (10)	0.0008 (7)	0.0009 (7)	0.0060 (8)
C13	0.0184 (9)	0.0207 (10)	0.0286 (10)	0.0021 (7)	0.0026 (8)	0.0045 (8)
C14	0.0162 (8)	0.0184 (9)	0.0253 (10)	0.0006 (7)	0.0031 (7)	0.0028 (8)
C15	0.0182 (9)	0.0233 (10)	0.0269 (10)	0.0020 (7)	0.0046 (8)	0.0046 (8)
C16	0.0180 (9)	0.0259 (10)	0.0276 (10)	0.0027 (7)	0.0054 (8)	0.0072 (8)
C17	0.0255 (10)	0.0383 (13)	0.0348 (12)	0.0085 (9)	0.0122 (9)	0.0145 (10)
C18	0.0271 (10)	0.0366 (12)	0.0342 (12)	0.0057 (9)	0.0107 (9)	0.0176 (10)
C19	0.0193 (9)	0.0267 (11)	0.0269 (10)	0.0006 (8)	0.0019 (8)	0.0100 (8)
C20	0.0239 (10)	0.0241 (10)	0.0301 (11)	0.0012 (8)	0.0044 (8)	0.0104 (8)
C21	0.0223 (10)	0.0250 (11)	0.0426 (13)	0.0054 (8)	0.0088 (9)	0.0136 (9)
C22	0.0416 (12)	0.0276 (11)	0.0370 (13)	0.0061 (9)	0.0097 (10)	0.0108 (10)
C23	0.0517 (14)	0.0334 (13)	0.0425 (14)	0.0108 (11)	0.0200 (12)	0.0186 (11)
C24	0.0356 (12)	0.0293 (12)	0.0700 (18)	0.0069 (10)	0.0256 (12)	0.0217 (12)
C25	0.0351 (13)	0.0434 (15)	0.079 (2)	−0.0141 (11)	−0.0025 (13)	0.0192 (15)
C26	0.0384 (13)	0.0436 (15)	0.0570 (17)	−0.0083 (11)	−0.0063 (12)	0.0244 (13)
C27	0.0236 (10)	0.0251 (11)	0.0527 (14)	0.0047 (8)	0.0123 (10)	0.0089 (10)
C28	0.0230 (10)	0.0322 (12)	0.0625 (17)	0.0016 (9)	0.0056 (10)	0.0138 (11)
C29	0.0254 (12)	0.0413 (15)	0.088 (2)	0.0031 (10)	0.0074 (13)	0.0234 (15)
C30	0.0276 (13)	0.0456 (16)	0.111 (3)	0.0123 (11)	0.0225 (16)	0.0168 (17)
C31	0.0514 (17)	0.0533 (18)	0.090 (2)	0.0129 (14)	0.0393 (18)	0.0002 (16)
C32	0.0391 (13)	0.0458 (15)	0.0619 (18)	0.0131 (11)	0.0208 (12)	0.0046 (13)
C33	0.0200 (9)	0.0228 (10)	0.0236 (10)	0.0022 (7)	0.0055 (7)	0.0042 (8)
C34	0.0314 (11)	0.0262 (11)	0.0280 (11)	−0.0021 (8)	0.0059 (9)	0.0012 (9)
C35	0.0350 (11)	0.0224 (10)	0.0452 (13)	−0.0018 (9)	0.0119 (10)	0.0069 (10)
C36	0.0421 (13)	0.0334 (12)	0.0390 (13)	0.0072 (10)	0.0204 (11)	0.0158 (10)
C37	0.0562 (15)	0.0418 (14)	0.0246 (11)	0.0057 (11)	0.0164 (10)	0.0042 (10)
C38	0.0453 (13)	0.0263 (11)	0.0277 (11)	−0.0007 (9)	0.0133 (10)	−0.0017 (9)
C39	0.0239 (9)	0.0275 (11)	0.0281 (11)	0.0083 (8)	0.0097 (8)	0.0113 (8)
C40	0.0327 (11)	0.0311 (12)	0.0427 (13)	0.0032 (9)	0.0134 (10)	0.0058 (10)
C41	0.0487 (14)	0.0331 (13)	0.0472 (15)	0.0129 (11)	0.0175 (12)	0.0027 (11)
C42	0.0369 (12)	0.0470 (15)	0.0422 (14)	0.0220 (11)	0.0192 (10)	0.0152 (11)
C43	0.0253 (11)	0.0476 (15)	0.0463 (14)	0.0098 (10)	0.0140 (10)	0.0161 (12)
C44	0.0248 (10)	0.0334 (12)	0.0402 (13)	0.0052 (9)	0.0090 (9)	0.0091 (10)
C45	0.0259 (10)	0.0345 (12)	0.0369 (12)	−0.0043 (9)	0.0007 (9)	0.0008 (10)
C46	0.0356 (13)	0.0486 (15)	0.0461 (15)	−0.0126 (11)	−0.0036 (11)	−0.0062 (12)
C47	0.0590 (17)	0.0426 (16)	0.0528 (17)	−0.0114 (13)	−0.0038 (14)	−0.0149 (13)
C48	0.0596 (17)	0.0450 (16)	0.0520 (17)	0.0046 (13)	0.0018 (13)	−0.0198 (13)
C49	0.0392 (13)	0.0342 (13)	0.0379 (13)	0.0034 (10)	0.0038 (10)	−0.0047 (10)
C9	0.0222 (9)	0.0196 (9)	0.0230 (10)	−0.0017 (7)	0.0063 (8)	−0.0003 (8)

### Geometric parameters (Å, º)

**Table:**

Co—N4	1.9498 (15)	C22—H22	0.9500
Co—N3	1.9564 (15)	C23—C24	1.365 (4)
Co—N2	1.9596 (16)	C23—H23	0.9500
Co—N1	1.9660 (16)	C24—C25	1.369 (4)
Co—N5	1.9898 (17)	C24—H24	0.9500
Co—Cl	2.2339 (6)	C25—C26	1.386 (3)
N1—C4	1.374 (2)	C25—H25	0.9500
N1—C1	1.375 (3)	C26—H26	0.9500
N2—C9	1.373 (2)	C27—C28	1.384 (3)
N2—C6	1.375 (2)	C27—C32	1.388 (4)
N3—C14	1.371 (2)	C28—C29	1.388 (3)
N3—C11	1.372 (2)	C28—H28	0.9500
N4—C19	1.361 (2)	C29—C30	1.357 (5)
N4—C16	1.377 (2)	C29—H29	0.9500
N5—C45	1.340 (3)	C30—C31	1.368 (5)
N5—C49	1.343 (3)	C30—H30	0.9500
C1—C20	1.385 (3)	C31—C32	1.395 (4)
C1—C2	1.431 (3)	C31—H31	0.9500
C2—C3	1.342 (3)	C32—H32	0.9500
C2—H2	0.9500	C33—C38	1.379 (3)
C3—C4	1.442 (3)	C33—C34	1.386 (3)
C3—H3	0.9500	C34—C35	1.384 (3)
C4—C5	1.384 (3)	C34—H34	0.9500
C5—C6	1.391 (3)	C35—C36	1.381 (3)
C5—C27	1.494 (3)	C35—H35	0.9500
C6—C7	1.434 (3)	C36—C37	1.368 (3)
C7—C8	1.343 (3)	C36—H36	0.9500
C7—H7	0.9500	C37—C38	1.388 (3)
C8—C9	1.436 (3)	C37—H37	0.9500
C8—H8	0.9500	C38—H38	0.9500
C10—C9	1.386 (3)	C39—C40	1.384 (3)
C10—C11	1.388 (3)	C39—C44	1.387 (3)
C10—C33	1.495 (3)	C40—C41	1.391 (3)
C11—C12	1.431 (3)	C40—H40	0.9500
C12—C13	1.344 (3)	C41—C42	1.374 (4)
C12—H12	0.9500	C41—H41	0.9500
C13—C14	1.432 (3)	C42—C43	1.378 (4)
C13—H13	0.9500	C42—H42	0.9500
C14—C15	1.392 (3)	C43—C44	1.382 (3)
C15—C16	1.383 (3)	C43—H43	0.9500
C15—C39	1.491 (3)	C44—H44	0.9500
C16—C17	1.434 (3)	C45—C46	1.373 (3)
C17—C18	1.345 (3)	C45—H45	0.9500
C17—H17	0.9500	C46—C47	1.371 (4)
C18—C19	1.427 (3)	C46—H46	0.9500
C18—H18	0.9500	C47—C48	1.381 (4)
C19—C20	1.390 (3)	C47—H47	0.9500
C20—C21	1.491 (3)	C48—C49	1.370 (4)
C21—C26	1.374 (3)	C48—H48	0.9500
C21—C22	1.382 (3)	C49—H49	0.9500
C22—C23	1.389 (3)		
			
N4—Co—N3	89.45 (6)	C22—C21—C20	123.0 (2)
N4—Co—N2	179.36 (7)	C21—C22—C23	120.3 (2)
N3—Co—N2	90.57 (6)	C21—C22—H22	119.8
N4—Co—N1	90.19 (6)	C23—C22—H22	119.8
N3—Co—N1	178.93 (7)	C24—C23—C22	120.5 (2)
N2—Co—N1	89.81 (6)	C24—C23—H23	119.7
N4—Co—N5	89.39 (7)	C22—C23—H23	119.7
N3—Co—N5	90.20 (7)	C23—C24—C25	119.6 (2)
N2—Co—N5	91.25 (7)	C23—C24—H24	120.2
N1—Co—N5	88.80 (7)	C25—C24—H24	120.2
N4—Co—Cl	89.09 (5)	C24—C25—C26	120.1 (3)
N3—Co—Cl	89.85 (5)	C24—C25—H25	120.0
N2—Co—Cl	90.27 (5)	C26—C25—H25	120.0
N1—Co—Cl	91.14 (5)	C21—C26—C25	121.0 (2)
N5—Co—Cl	178.48 (5)	C21—C26—H26	119.5
C4—N1—C1	105.72 (16)	C25—C26—H26	119.5
C4—N1—Co	127.59 (13)	C28—C27—C32	118.8 (2)
C1—N1—Co	126.20 (13)	C28—C27—C5	120.8 (2)
C9—N2—C6	106.02 (15)	C32—C27—C5	120.3 (2)
C9—N2—Co	126.47 (12)	C27—C28—C29	120.5 (3)
C6—N2—Co	126.91 (13)	C27—C28—H28	119.8
C14—N3—C11	105.46 (15)	C29—C28—H28	119.8
C14—N3—Co	127.58 (13)	C30—C29—C28	120.3 (3)
C11—N3—Co	126.90 (12)	C30—C29—H29	119.9
C19—N4—C16	106.04 (16)	C28—C29—H29	119.9
C19—N4—Co	126.75 (13)	C29—C30—C31	120.4 (2)
C16—N4—Co	126.76 (13)	C29—C30—H30	119.8
C45—N5—C49	117.70 (19)	C31—C30—H30	119.8
C45—N5—Co	120.95 (15)	C30—C31—C32	120.3 (3)
C49—N5—Co	121.26 (15)	C30—C31—H31	119.9
N1—C1—C20	125.32 (18)	C32—C31—H31	119.9
N1—C1—C2	110.37 (17)	C27—C32—C31	119.8 (3)
C20—C1—C2	124.17 (19)	C27—C32—H32	120.1
C3—C2—C1	106.89 (19)	C31—C32—H32	120.1
C3—C2—H2	126.6	C38—C33—C34	118.77 (19)
C1—C2—H2	126.6	C38—C33—C10	121.28 (18)
C2—C3—C4	107.48 (18)	C34—C33—C10	119.82 (18)
C2—C3—H3	126.3	C35—C34—C33	120.8 (2)
C4—C3—H3	126.3	C35—C34—H34	119.6
N1—C4—C5	125.60 (18)	C33—C34—H34	119.6
N1—C4—C3	109.50 (17)	C36—C35—C34	119.8 (2)
C5—C4—C3	124.66 (18)	C36—C35—H35	120.1
C4—C5—C6	122.57 (18)	C34—C35—H35	120.1
C4—C5—C27	118.54 (18)	C37—C36—C35	119.8 (2)
C6—C5—C27	118.63 (18)	C37—C36—H36	120.1
N2—C6—C5	125.57 (18)	C35—C36—H36	120.1
N2—C6—C7	109.55 (17)	C36—C37—C38	120.5 (2)
C5—C6—C7	124.75 (17)	C36—C37—H37	119.8
C8—C7—C6	107.47 (17)	C38—C37—H37	119.8
C8—C7—H7	126.3	C33—C38—C37	120.3 (2)
C6—C7—H7	126.3	C33—C38—H38	119.8
C7—C8—C9	107.06 (17)	C37—C38—H38	119.8
C7—C8—H8	126.5	C40—C39—C44	118.96 (19)
C9—C8—H8	126.5	C40—C39—C15	119.36 (18)
C9—C10—C11	122.29 (17)	C44—C39—C15	121.67 (19)
C9—C10—C33	117.61 (16)	C39—C40—C41	120.2 (2)
C11—C10—C33	119.71 (17)	C39—C40—H40	119.9
N3—C11—C10	125.43 (17)	C41—C40—H40	119.9
N3—C11—C12	110.17 (16)	C42—C41—C40	120.3 (2)
C10—C11—C12	123.88 (17)	C42—C41—H41	119.8
C13—C12—C11	106.99 (17)	C40—C41—H41	119.8
C13—C12—H12	126.5	C41—C42—C43	119.8 (2)
C11—C12—H12	126.5	C41—C42—H42	120.1
C12—C13—C14	107.08 (16)	C43—C42—H42	120.1
C12—C13—H13	126.5	C42—C43—C44	120.2 (2)
C14—C13—H13	126.5	C42—C43—H43	119.9
N3—C14—C15	125.33 (17)	C44—C43—H43	119.9
N3—C14—C13	110.14 (16)	C43—C44—C39	120.5 (2)
C15—C14—C13	124.50 (17)	C43—C44—H44	119.7
C16—C15—C14	122.09 (17)	C39—C44—H44	119.7
C16—C15—C39	119.04 (17)	N5—C45—C46	122.7 (2)
C14—C15—C39	118.82 (17)	N5—C45—H45	118.6
N4—C16—C15	125.07 (18)	C46—C45—H45	118.6
N4—C16—C17	109.39 (17)	C47—C46—C45	119.0 (2)
C15—C16—C17	124.97 (18)	C47—C46—H46	120.5
C18—C17—C16	106.99 (18)	C45—C46—H46	120.5
C18—C17—H17	126.5	C46—C47—C48	118.9 (2)
C16—C17—H17	126.5	C46—C47—H47	120.5
C17—C18—C19	107.18 (18)	C48—C47—H47	120.5
C17—C18—H18	126.4	C49—C48—C47	119.0 (3)
C19—C18—H18	126.4	C49—C48—H48	120.5
N4—C19—C20	126.24 (18)	C47—C48—H48	120.5
N4—C19—C18	110.18 (17)	N5—C49—C48	122.6 (2)
C20—C19—C18	123.39 (18)	N5—C49—H49	118.7
C1—C20—C19	121.87 (18)	C48—C49—H49	118.7
C1—C20—C21	119.86 (18)	N2—C9—C10	126.52 (17)
C19—C20—C21	118.27 (18)	N2—C9—C8	109.82 (17)
C26—C21—C22	118.4 (2)	C10—C9—C8	123.58 (18)
C26—C21—C20	118.6 (2)		

### Hydrogen-bond geometry (Å, º)

Cg2, Cg3, Cg6, Cg9, Cg11 and Cg12 are the centroids of the N2/C6–C9, N3/C11–C14, Co/N1/C4–C6/N2, N5/C45–C49, C27–C32 and C33–C38 rings, respectively.

**Table:**

*D*—H···*A*	*D*—H	H···*A*	*D*···*A*	*D*—H···*A*
C24—H24···*Cg*3^i^	0.95	2.79	3.543 (3)	137
C28—H28···*Cg*9^ii^	0.95	2.79	3.735 (3)	172
C35—H35···*Cg*2^iii^	0.95	2.87	3.736 (2)	152
C38—H38···*Cg*11^iv^	0.95	2.98	3.861 (3)	156
C42—H42···*Cg*12^v^	0.95	2.75	3.574 (3)	146
C49—H49···*Cg*6	0.95	2.35	2.931 (3)	119

Symmetry codes: (i) −*x*+3/2, *y*+1/2, −*z*+3/2; (ii) *x*−3/2, −*y*−1/2, *z*−3/2; (iii) −*x*+1, −*y*, −*z*+2; (iv) *x*−1/2, −*y*−1/2, *z*−1/2; (v) −*x*+2, −*y*, −*z*+2.
